# Outdoor work as risk factor for facial melanoma: UV exposure, risk awareness, and occupational relevance

**DOI:** 10.1111/ddg.15995

**Published:** 2026-01-21

**Authors:** Susanne Dugas‐Breit, Jessica C. Hassel, Martin Dugas, Hans‐Joachim Schulze

**Affiliations:** ^1^ Department of Dermatology and National Center for Tumor Diseases (NCT) NCT Heidelberg a partnership between DKFZ and University Hospital Heidelberg Heidelberg University Heidelberg Germany; ^2^ Institute of Medical Informatics Heidelberg University Hospital Heidelberg Germany; ^3^ Department of Dermatology Fachklinik Hornheide Münster Germany

**Keywords:** Melanoma, occupational UV exposure, preventive measures, risk awareness

## Abstract

**Background:**

This study explored UV exposure and prevention behaviors among melanoma patients, focusing on occupational UV exposure, melanoma characteristics, risk awareness, and protective behaviors.

**Patients and Methods:**

This cross‐sectional study analyzed demographics, melanoma characteristics, UV exposure, awareness, and preventive measures. Data were stratified by occupational exposure (indoor vs. outdoor) and analyzed using logistic regression to identify predictors for melanoma location.

**Results:**

Among 406 patients (54% female; median age 57), 59 (15%) had a history of outdoor work. Outdoor workers were more likely to develop melanoma in sun‐exposed areas. They also had a higher prevalence of facial melanoma (p = 0.020); those with facial involvement had worked outdoors twice as long as those with melanoma at other sites, indicating a dose‐effect relationship. Logistic regression identified outdoor work (OR 2.47) and age (OR 1.05) as significant predictors of facial melanoma. A total of 229 patients (57%) were unaware of the potential harms of UV radiation before diagnosis. Among outdoor workers, only 3 (5%) reported frequent sun protection, while 33 (56%) used it rarely or never.

**Conclusions:**

Outdoor work is a significant risk factor for facial melanoma in this cohort. Sun protection and UV awareness remain insufficient, particularly among outdoor workers, highlighting the need for targeted prevention.

## INTRODUCTION

Solar ultraviolet (UV) radiation is regarded as the most significant environmental risk factor for the development of melanoma. The incidence of melanoma has been rising globally, with UV radiation from both natural and artificial sources playing a central role in its development. The relationship between UV exposure and non‐melanoma keratinocyte skin cancer is well‐documented,^1^, ^2^ leading to the designation of cutaneous squamous cell carcinoma caused by occupational natural UV exposure as an occupational disease in Germany in 2015.^3^ However, the evidence linking occupational UV exposure specifically to melanoma remains equivocal.^4^, ^5^, ^6^, ^7^, ^8^ This inconsistency is partly due to the multifactorial nature of melanoma pathogenesis, which involves genetic predisposition, intermittent UV exposure, and chronic cumulative UV damage.^9^, ^10^

Around 12% of workers in Germany are employed in jobs that are carried out exclusively or predominantly outdoors, such as construction, agriculture, forestry, horticulture and other outdoor activities.^11^ Approximately 7.2 million employees in Germany regularly spend more than 22% of their working time outdoors or receive more than 150 standard erythema doses of UV radiation per year.^12^ The annual exposure of this group of employees to UV radiation is, on average, up to three times higher than that of employees who do not work outdoors.^13^ Wittlich's measurements indicate that the limit value proposed by the WHO for UV exposure in the workplace is regularly exceeded by a factor of five. This is not the case with any other carcinogen.^14^, ^15^

The conventional classification system for melanoma is based on histological growth patterns, with division into four main subtypes: superficial spreading, lentigo maligna, nodular, and acral lentiginous.^16^, ^17^, ^18^ The lentigo maligna melanoma (LMM) subtype, which accounts for 5 to 15% of all melanomas, occurs almost exclusively in chronically sun‐exposed areas of the body.^19^, ^20^, ^21^, ^22^ This indicates a clear association with chronic (cumulative) UV exposure. Nevertheless, there are currently no epidemiological studies that provide sufficient evidence that any group of people is at significantly higher risk of developing LMMs due to occupational exposure (chronic, cumulative UV radiation).^7^, ^13^, ^21^, ^22^

Despite the known risks, the use of preventive measures among outdoor workers remains inadequate. Previous studies have indicated low awareness of UV risks and insufficient use of protective measures such as sunscreen, protective clothing, or shade‐seeking behaviors.^23^, ^24^, ^25^ A survey conducted by the *Federal Institute for Occupational Safety and Health* in 2019 revealed that only a third of outdoor workers in Germany reported receiving regular instruction on the dangers of solar radiation.^11^ In addition, standardized tools for assessing UV exposure awareness and sun safety behaviors, particularly among outdoor workers, were lacking before this study.

This study aims to address these gaps by examining occupational UV exposure, the prevalence and characteristics of melanoma subtypes, and the awareness and adoption of preventive measures in a cohort of melanoma patients.

## METHODS AND PATIENTS

### Study design

A single‐center, cross‐sectional study was conducted at the Department of Dermatology at Fachklinik Hornheide. Patients aged ≥ 18 diagnosed with stage 0 to IV melanoma were eligible for inclusion, with staging based on 2017 AJCC guidelines.^26^ Exclusions applied to patients lacking German language skills or those unable to complete the questionnaires independently. Between 2019 and 2021, patients with informed consent completed an electronic survey and a clinical routine assessment. The study was registered with the German Clinical Trials Register (DRKS00016939).

### Survey instruments and data management

Electronic surveys gathered data on demographics, occupational background, recreational or occupational UV exposure, history of sunburns as child and adult, awareness of UV radiation risks, and preventive behavior. Occupational background included detailed information on the economic sector of employment, current work status (e.g., full‐time, part‐time, retired), and whether the patient worked predominantly indoors or outdoors. Participants were classified as outdoor workers if they reported regularly working outdoors. All others were assigned to the indoor group, regardless of current employment status, based on the absence of regular outdoor occupational activity. To assess UV‐related risk awareness, participants were asked the following question: “Did you know (in the past) that sunlight can be harmful?” (response options: yes/no). This self‐reported item was used to operationalize UV awareness. All questions used predefined categorical answer options (e.g., “never”, “rarely”, “sometimes”, “frequently”) The skin type according to Fitzpatrick was determined using a standardized questionnaire.^27^ The complete questionnaire is included in the online supplement. The selected survey items were adapted from established sources, including the Work Ability Index and the FB‐181 population‐based study.^1^, ^28^ In addition, data were collected on the subtype, location and stage of the melanoma, using the international classification of diseases for oncology system (ICD‐O‐3, 2nd Edition 2019).^29^ The results of this study are reported in accordance with the *Strengthening the Reporting of Observational Studies in Epidemiology* (STROBE) guidelines.^30^

### Statistics

This was a cross‐sectional study. Electronically recorded study data were checked for completeness and plausibility. 406 out of 414 patients provided evaluable data (for sample size estimation and Strobe flow chart see online supplement). Descriptive statistics were performed for all clinical parameters and survey forms on UV exposure and occupational history. Data were analyzed using the statistical program R (version 4.1.4., ggplot2 library). Statistical tests for associations between variables were based on a two‐sided significance level of 5%. Regarding missing values, no imputation was performed. Non‐parametric tests were used (no assumption of normally distributed data), specifically Wilcoxon tests (unpaired or paired). Pearson's chi‐squared Test or exact Fisher test was applied to analyze tabular data. Multivariable logistic regression was performed to assess significance of the following 15 predictor variables: Sex, age, body mass index, melanoma in the family, education, outdoor versus indoor worker, sun protection for work, artificial UV exposure (leisure/occupational), solarium use, leisure UV exposure, sun protection for leisure, sunburns as child, sunburn as adult, field carcinomatosis, and skin type according to Fitzpatrick were analyzed as predictors for facial melanoma. Predictor variables with a p value < 0.05 in univariable logistic regression were included in a forward selection procedure to build the multivariable model. There was no adjustment for multiple testing (non‐confirmatory observational study).

### Ethical approval

This study was conducted in accordance with the principles of the Declaration of Helsinki. Approval was obtained from the Ethics Committee Westfalen‐Lippe (reference number 2019‐258‐f‐S). All patients included in this manuscript provided written informed consent for the publication of their case details.

## RESULTS

### Characteristics and occupational situation of patients

The study cohort consisted of 406 patients aged 21–89 years, of whom 219 (54%) were female. Further demographic and clinical details are presented in Table 1. Melanoma characteristics and staging are shown in Table 2. The median tumor thickness (Breslow, AJCC 2017) was 1.2 mm (range 0.1–35 mm; IQR 0.7–2.4 mm).

**TABLE 1:** Patient population characteristics.^*^

	Total n (%)	Indoor	Outdoor	p value
**All Patients**	406	343 (85%)	59 (15%) ^**^	
Female	219 (54%)	205 (60%)	12 (20%)	< 0.001
Male	187 (46%)	138 (40%)	47 (80%)	
***Median Age in years (range)***				
All Patients	57 (21‐89)	56	58	0.24
Female	56 (22‐89)	54	57.5	
Male	58 (21‐89)	58	58	
***Skin Type (Fitzpatrick)***				0.25
I	6 (2%)	6 (2%)	0 (0%)	
II	198 (51%)	172 (51%)	24 (41%)	
III	186 (45%)	152 (45%)	33 (56%)	
IV	9 (2%)	7 (2%)	2 (3%)	
V	0 (0%)	0 (0%)	0 (0%)	
VI	0 (0%)	0 (0%)	0 (0%)	
***Body mass index (BMI) (kg/m^2^)***				
Median (range)	26.9 (16.6‐47.8)	26.4	29.4	<0.001
***Education*** ^**^				< 0.001
≤ 8 Years	148 (37%)	110 (32%)	36 (61%)	
10 Years	122 (30%)	111 (32%)	11 (19%)	
Baccalaureate	90 (22%)	82 (24%)	8 (14%)	
University Degree	44 (11%)	39 (11%)	4 (7%)	
***Work***				0.001
Full‐time	216 (53%)	174 (51%)	40 (68%)	
Part‐time	80 (20%)	77 (22%)	3 (5%)	
Unemployed	28 (7%)	27 (8%)	1 (2%)	
Pensioner	81 (20%)	65 (19%)	15 (25%)	
***Family history of melanoma***				0.37
No	330 (82%)	275 (81%)	51 (86%)	
Yes	72 (18%)	64 (19%)	8 (14%)	

* Some Percentages do not add up to 100% due to rounding.

** Four patients did not provide any information about indoor or outdoor work.

**TABLE 2:** Melanoma characteristics.

		Total n (%)	Indoor	Outdoor	p value
**Total**		406	343	59^*^	
***Type***	Superficial spreading	196 (48%)	167 (49%)	29 (49%)	0.92
	Nodular	54 (13%)	44 (13%)	10 (17%)	0.44
	Lentigo maligna or Lentigo maligna‐melanoma	48 (12%)	38 (11%)	7 (12%)	>0.99
	Acrolentiginous	18 (4%)	16 (5%)	1 (2%)	0.50
	Amelanotic	5 (1%)	5 (1%)	0 (0%)	>0.99
	In‐situ‐melanoma	11 (3%)	10 (3%)	1 (2%)	>0.99
	Unspecified or other	74 (18%)	63 (18%)	11 (19%)	>0.99
***Location***	Lower extremities and hip	111 (27%)	101 (29%)	10 (17%)	0.11
	Torso	132 (33%)	110 (32%)	22 (37%)	0.54
	Upper extremities and shoulder	79 (19%)	69 (20%)	10 (17%)	0.75
	Face	45 (11%)	31 (9%)	12 (20%)	0.033
	Head and Neck	26 (6%)	21 (6%)	3 (5%)	>0.99
	Unknown	11 (3%)	9 (3%)	2 (3%)	0.67
	Genital	2 (1%)	2 (1%)	0 (0%)	>0.99
**Melanoma stage**	0	29 (7%)	22 (6%)	5 (8%)	0.81
	I	202 (50%)	182 (54%)	19 (32%)	0.030
	II	75 (19%)	58 (17%)	16 (27%)	0.16
	III	82 (20%)	66 (19%)	16 (27%)	0.29
	IV	14 (3%)	11 (3%)	3 (5%)	0.74

* Four patients did not provide any information about indoor or outdoor work.

A total of 59 (15%) patients reported that they had regularly worked outside, with a median duration of 25 years (range 3–52, IQR 14–40). Of them, 47 (80%) were male. There were significantly more men in the outdoor group than the indoor group (n = 138; 40%) (p < 0.001). Furthermore, the group of outdoor workers showed a higher body mass index, lower educational attainment, and a higher proportion of full‐time employment. Information on the reported economic sector of employment is provided in online supplementary Table S1.

### UV exposure of patients

Data on UV exposure characteristics of all patients are summarized in Table 3. Time spent outdoors during leisure varied, with 78% of patients being outside (almost) daily or often. A total of 59% reported having visited solariums at some point, and childhood or adult sunburns were common. Among all patients, occupational or private exposure to artificial UV radiation (e.g., welding) was reported by 15%. Field carcinomatosis of sun‐exposed skin as a result of chronic UV damage was present in 12% of patients. No significant difference was found between indoor and outdoor workers (p = 0.83).

**TABLE 3:** UV exposure characteristics of all patients.^*^

	Total n (%)	Indoor	Outdoor	p value
***Time spent outdoors (leisure)***				
(Almost) daily	115 (28%)	83 (24%)	31 (53%)	<0.001
Often	194 (48%)	170 (50%)	22 (38%)	0.23
Occasionally	88 (22%)	83 (24%)	5 (9%)	0.019
(Almost) never	3 (1%)	3 (1%)	0 (0%)	>0.99
***Solarium use***				
Never	166 (41%)	119 (35%)	43 (73%)	<0.001
Few times in life	160 (40%)	148 (43%)	12 (20%)	0.007
Several times	52 (13%)	49 (14%)	3 (5%)	0.08
(Almost) annually	4 (1%)	4 (1%)	0 (0%)	>0.99
Regularly	24 (6%)	23 (7%)	1 (2%)	0.16
***Artificial UV exposure (occupational)***				
Yes	11 (3%)	8 (2%)	3 (5%)	0.21
No	395 (97%)	332 (98%)	55 (95%)	
***Artificial UV exposure (private)***				
Yes	49 (12%)	42 (12%)	6 (10%)	0.83
No	357 (88%)	298 (88%)	52 (90%)	
***Sunburns in childhood (< 18 years)***				
Never	24 (6%)	22 (6%)	2 (3%)	0.57
A few times	168 (41%)	132 (39%)	34 (58%)	0.049
Several times in total	97 (24%)	89 (26%)	7 (12%)	0.042
(Almost) annually	77 (19%)	71 (21%)	6 (10%)	0.11
Several per year	39 (10%)	28 (8%)	10 (17%)	0.06
***Sunburns in adulthood***				
Never	21 (5%)	17 (5%)	4 (7%)	0.54
A few times	187 (46%)	158 (46%)	26 (44%)	0.92
Several times in total	120 (30%)	106 (31%)	14 (24%)	0.44
(Almost) annually	55 (14%)	48 (14%)	6 (10%)	0.57
Several per year	22 (5%)	13 (4%)	9 (15%)	0.003
***Field carcinomatosis***				
Yes	50 (12%)	42 (12%)	8 (14%)	0.83
No	355 (88%)	300 (88%)	51 (87%)	

* Some Percentages do not add up to 100% due to rounding.

### Association of melanoma location with UV‐exposure

A total of 84 (21%) patients stated that their melanoma was located in a body region that was always exposed to sunlight, while 150 (37%) patients indicated that the location was often exposed to sunlight, depending on the season. In 141 (35%) patients, the melanoma was rarely exposed to sunlight, and in 27 (7%) patients, it was never exposed to sunlight.

Face melanoma site was significantly associated with outdoor worker status (odds ratio 2.56, 95% confidence interval 1.12–5.57; p = 0.020), i.e., outdoor workers were more likely to have a melanoma in the face compared to indoor workers (Table 2).

Outdoor workers with a melanoma in the facial region had worked outdoors a median of 40 years, while outdoor workers with a melanoma elsewhere had worked outdoors a median of 20 years (p = 0.015). Among outdoor workers, melanoma was significantly more common in areas always or often exposed to the sun (n = 39; 66%) than among indoor workers (n = 192; 56%; p = 0.001). These findings suggest a dose‐effect relationship for melanoma in the face region among outdoor workers.

Female patients demonstrated a significantly higher frequency of melanoma in the lower extremities compared to male patients (n = 78; 70% vs. n = 33; 30%; p < 0.001). This observation accounts for the non‐significant difference (p = 0.07) in the distribution of this specific melanoma localization between the indoor and outdoor groups (Table 2). Furthermore, women exhibited a higher likelihood of being diagnosed with stage I melanoma compared to men (n = 123; 61% vs. n = 79; 39%; p = 0.003). This observation, once again, accounts for the significant difference (p = 0.004) in the distribution of stage I melanoma observed between the indoor and outdoor groups (Table 2).

Regarding outdoor recreational activities, there was no significant association between frequency of UV exposure and melanoma location (Fishers exact test p = 0.40), and no monotonic association (spearman correlation 0.038; p = 0.45). There was also no association between histological melanoma type and outdoor/indoor work (p = 0.98).

Multivariable logistic regression with forward selection was performed to identify predictors for facial melanoma (Details of the univariable logistic regression analysis are provided in online supplementary Table S2):

The adjusted multivariable analysis revealed that outdoor worker status (OR 2.47, 95% CI 1.13–5.15; p = 0.018) and age (OR 1.05, 95% CI 1.02–1.07; p<0.001) were the only two significant risk factors for developing facial melanoma.

### Sun protection behaviors

A total of 125 (31%) patients indicated that they frequently used sun protection during their leisure time, 166 (41%) sometimes, 95 (24%) rarely, and 18 (5%) never. In the group of patients who worked outdoors, 15 (25%) reported never using sun protection (like sun screen or protective clothing), 18 (31%) rarely used it, 23 (39%) sometimes used it, and only 3 (5%) frequently used sun protection.

In response to the inquiry regarding their awareness of the potential harms of sunlight, a total of 229 (57%) patients indicated a lack of awareness prior to the diagnosis of melanoma. Our study revealed no statistically significant discrepancy between indoor and outdoor workers with respect to their awareness of the potential harms of UV radiation (p = 0.21).

## DISCUSSION

This study highlights the significant role of occupational UV exposure in the development of facial melanoma, underlining its relevance as an occupational disease. Our analysis of 406 patients shows that outdoor workers have a significantly higher risk of developing facial melanoma compared to indoor workers (OR 2.47). In 2021, the *Cancer Registry Baden‐Württemberg* reported an incidence of 8.1% for facial melanoma among all patients,^31^ which is consistent with the incidence among indoor workers in our patient population. These results align with previous research suggesting a strong association between chronic UV exposure and melanoma in the head and neck area, which is frequently exposed to sunlight during outdoor activities.^32^, ^33^, ^34^, ^35^, ^36^ In fact, excess risk has been reported in occupations such as farming,^37^ or construction.^38^

Furthermore, a significant dose‐effect relationship was identified: In comparison to indoor workers, outdoor workers were significantly more likely to develop melanoma in areas always or often exposed to the sun. In addition, outdoor workers who developed facial melanoma worked outside twice as long as outdoor workers who developed melanoma elsewhere. Furthermore, no statistically significant association was found between leisure‐time UV exposure and the incidence of facial melanoma, suggesting that occupational UV exposure outweighs recreational UV exposure in contributing to risk. While most studies categorized melanoma by histological subtype,^17^, ^39^, ^40^ future research should emphasize anatomical site‐specific risks. We did not find a statistically significant accumulation of field carcinogenesis as a sign of long‐term chronic UV exposure in outdoor workers of our cohort compared to indoor workers, which is in line with different mutational profiles of melanoma in chronically sun‐exposed versus intermittently exposed skin.^9^, ^36^ This is consistent with others who have suggested the existence of distinct pathways that may be related to anatomical location.^41^, ^42^

With respect to the implementation of preventive measures, this study found an alarming lack of sun protection strategies to minimize occupational UV exposure in the cohort studied: Despite higher UV exposure, only 5% of outdoor workers reported regular use of sun protective measures during occupational activities, and 56% never or rarely used such measures. In comparison, sunscreen use was more common in the recreational setting among our patients, highlighting the need for targeted educational initiatives in the professional setting. These findings are in line with previous research,^43^ which found that 70% of outdoor workers felt inadequately protected from UV exposure.

It is difficult to assess whether melanoma patients have a more frequent history of sunburn than the rest of the population due to a lack of comparative data. Our melanoma patients appear to have similar sunburn histories to the general population: In a 2016 survey, 41% of adult respondents reported having had at least one sunburn in the past year.^44^ Regarding solarium use, our melanoma patient cohort showed a markedly higher prevalence compared to the general population. While 27% of our patients reported frequent or regular solarium visits, only 9% did so in a 2007 survey.^45^ More recent data from a representative German population study between 2015 and 2022 indicate a further decline, with current tanning bed use reported at only 5%–6%.^46^ Therefore, despite advances in preventive strategies, including behavioral interventions and sunscreen campaigns,^47^, ^48^ the adoption of sun safety practices remains insufficient.

Our findings also reveal a significant knowledge gap: 57% of all patients (229 of 406) reported that they had been unaware prior to their melanoma diagnosis that sunlight can be harmful. Consequently, there is an urgent need for ongoing education and training in this area, particularly in the context of vocational training and in the workplace.

In recent years, there has been an increase in efforts to address these deficiencies. In particular, the development of standardized measurement tools, such as the *Sun Exposure and Protection Index* ^49^ and the *Skin Cancer and Sun Knowledge Scale* ^50^ provide valuable instruments for assessing sun safety behaviors and knowledge. These tools, along with validated translated questionnaires,^51^ should be used more widely, especially in occupational settings, to identify risky behaviors and provide tailored prevention strategies.

Our study has several strengths: It is based on a cohort of meaningful size with high level of data completeness, covering comprehensive patient information and standardized medical examinations. Several findings from cancer registry data (e.g., melanoma being more frequent on the legs in women) are reflected in our results, further supporting the quality and validity of the data.

The limitations of this study must be taken into consideration when interpreting the results: This is a single‐center cohort study with cross‐sectional data. Therefore, our findings may not be fully generalizable. Dosimetry data concerning occupational and recreational UV exposure is not available. Consequently, the assessment is compelled to rely on patient information, which may limit the precision of exposure‐risk correlations. Furthermore, the combined questionnaire used in this study was not formally validated as a whole, which represents a methodological limitation. However, it was based on previously applied and peer‐reviewed instruments, such as the *Work Ability Index* and the FB‐181 population‐based study.^1^, ^28^ A further problem is selection bias, as only patients with higher tumor stages regularly attend medical follow‐up examinations and were therefore slightly overrepresented in our survey compared to other sources of reported melanoma cases.^52^

In conclusion, facial melanoma was more common among outdoor workers and associated with longer durations of occupational sun exposure, suggesting a potential occupational relevance similar to that established for squamous cell carcinoma. Significant gaps in awareness and preventive behaviors were identified, highlighting the ongoing critical need to include education on the importance of UV protection measures in the vocational training of all professions working outdoors, especially in times of increasing UV exposure.^53^ To ensure compliance with these measures, periodic evaluations should be conducted as an integral part of professional activities.

## FUNDING INFORMATION

The study was funded by a grant from *Deutsche Rentenversicherung* (grant id 622‐4023). The funder had no role in the design, data collection, data analysis, and reporting of this study.

## CONFLICT OF INTEREST STATEMENT

J.C.H. receives honoraria from BMS, Immunocore, MSD, Novartis, Philogen, Pierre Fabre and Sanofi as a speaker, and from Onkowissen and Sunpharma as a participant in advisory boards. S.D.B., M.D. and H.J.S. declare no conflict of interest.

## Supporting information



Supplementary information
