# Supplementary material for: Outdoor work as risk factor for facial melanoma: UV exposure, risk awareness, and occupational relevance
Source: J Dtsch Dermatol Ges. 2026 Jan 21;24(9):1232–9. doi: 10.1111/ddg.15995 (PMC13551220; doi:10.1111/ddg.15995)
Supplement: Supplementary file 1 [file DDG-24-1232-s001.docx]

**Online Supplementary Material**

Outdoor Work as a Risk Factor for Facial Melanoma:
UV Exposure, Risk Awareness, and Occupational Relevance in 406 Patients

Susanne Dugas-Breit, Jessica C. Hassel, Martin Dugas^*^, Hans-Joachim Schulze^*^

* These authors share last authorship.

Corresponding Author:
Susanne Dugas-Breit
Department of Dermatology, Heidelberg University Hospital, Heidelberg, Germany
susanne.dugas-breit@med.uni-heidelberg.de

**Sample size estimation**

Sample size was estimated with the following assumption:
15 predictors should be analyzed with logistic regression, 10 events per predictor and an event rate of 0.4.
This results in 375 patients. To account for up to 10% patients with incomplete data, a total sample size of 413 was planned.

STROBE Flow Chart

Assessed for Eligibility: n=414

unwilling to complete the questionnaire:
- due to time reasons: n=4
- without giving reasons: n=2

Given consent: n=408

Questionnaire completed twice: n=2

Complete Dataset: n=406

**Table S1** Economic sector in which patients are/were employed

| **Economic sector** | **Number of patients** |
| --- | --- |
| Agriculture, forestry and fishing | 50 (12%) |
| Manufacturing industry excluding construction | 29 (7%) |
| Construction | 3 (1%) |
| Trade, gastronomy, transportation | 87 (21%) |
| Financing, real estate, corporate service providers | 22 (5%) |
| Public and private services | 178 (44%) |
| Other | 37 (9%) |

**Table S2**

Univariable logistic regression analysis for the association between each individual predictor and the outcome "facial melanoma" (no adjustment for other predictors). Odds ratios (OR) are presented along with 95% confidence intervals (CI), and associated p-values.

| Variable | OR | 95% CI OR | p-value |
| --- | --- | --- | --- |
| sex | 1.19 | 0.639 – 2.26 | 0.584 |
| age | 1.05 | 1.02 – 1.07 | <0.001* |
| BMI | 0.985 | 0.925 – 1.04 | 0.630 |
| melanoma family history | 1.17 | 0.506 – 2.45 | 0.698 |
| education | 0.349 | 0.134 – 0.809 | 0.020* |
| outdoor | 2.57 | 1.20 – 5.25 | 0.012* |
| sunprotection for work | 0.421 | 0.086 – 1.93 | 0.265 |
| artificial UV exposure | 0.669 | 0.194 – 1.76 | 0.463 |
| solarium use | 0.546 | 0.260 – 1.10 | 0.099 |
| leisure UV exposure | 1.48 | 0.656 – 3.38 | 0.340 |
| sun protection for leisure | 0.399 | 0.126 – 1.53 | 0.140 |
| Sunburns in childhood (<18 years) | 0.423 | 0.110 – 1.79 | 0.214 |
| Sunburns in adulthood | 1.76 | 0.374 – 9.69 | 0.480 |
| field carcinomatosis | 0.514 | 0.239 – 1.20 | 0.103 |
| skin type | 0.825 | 0.431 – 1.56 | 0.554 |
|  |  |  |  |

**Questionnaire (translated from German)**

**What is your height?**

__________ (in cm)

**What is your weight?**

__________ (in kg)

**Does your family have a history of melanoma?**

	Yes

	No

**What is your level of education?**

□ Still at school

□ No school leaving certificate

□ Primary/secondary school certificate/ 8th grade Polytechnic high school

□ Secondary education/ intermediate education/ 10th grade Polytechnic high school

□ Technical baccalaureate (entrance qualification for universities of applied sciences)

□ Baccalaureate / higher education entrance qualification/ Extended secondary school

□ Completed university degree

□ Other school leaving qualification

**Which of the following describes your employment situation before you were diagnosed with melanoma?**

 Working full time

 Working at least half the day

 Working less than half a day

 Retired due to reduced earning capacity / old age

 Housewife/-husband

 Not gainfully employed for other reasons

 unemployed

**In which sector of the economy can your (former) job be classified? (If you have had more than one job, please select the one in which you have worked the longest).**

 Agriculture and forestry

 Fishing and fish farming

 Mining and quarrying

 Manufacturing

 Electricity, gas and water supply

 Construction

 Wholesale and retail trade; repair of motor vehicles, personal and household goods

 Hotels and restaurants

 Transportation; communication

 Financial intermediation

 Real estate; renting, business activities

 Public administration; defence; compulsory social security

 Education

 Health; social work; Veterinary services

 Other community, social and personal service activities

 Private households

 Extra-territorial organisations and bodies

 Manufacturing without further specification or not assignable

 Services without further specification or not assignable

 Not assignable

**Did you regularly work outdoors (e.g. as a gardener, construction worker, etc.)?**

 Yes

 No

***if yes:* How many years have you regularly worked outdoors?**

____ years

***if yes:*
Have you used sun protection (e.g. sun cream, sun protective clothing) at work?**

 Never

 Rarely

 Sometimes

 Frequently

**Have you been exposed to *artificial* UV radiation?**

 Yes, occupational (e.g. through welding)

 Yes, private (through hobbies)

 No

**Have you ever been to a solarium?**

 Never

 A few times

 Several times

 (Almost) annually

 Regularly

**Is your melanoma located in an area of the body that has been exposed to sunlight?**

 Always

 Often, depending on the season

 Rarely

 Never

**How would you rate your outdoor leisure behavior?**

 Outside (almost) every day

 Often outside

 Outside from time to time

 (Almost) never outside

**Did you use sun protection (e.g. sun cream, sun protective clothing) during your leisure time?**

 Never

 Rarely

 Sometimes

 Frequently

**How many sunburns (including “just” reddening of the skin) did you have BEFORE the age of 18?**

 Never

 Some

 (Almost) annually

 Several per year

**How many sunburns (including “just” reddening of the skin) did you have AFTER the age of 18?**

 Never

 Some

 (Almost) annually

 Several per year

**Did you know (in the past) that sunlight can be harmful?**

 Yes

 No

**How is your general state of health?**

 Excellent

 Very good

 Good

 Less good

 Poor

**Now a few more questions about your skin type:**

**What color is your skin (without sun)?**

 Reddish

 Whitish

 Slightly beige

 Brownish

**Does your skin have freckles?**

 Yes, many

 Yes, some

 Yes, but a few

 No

**How does your facial skin react to the sun?**

 Very sensitive, mostly skin tension

 Sensitive, sometimes skin tension

 Normally sensitive, only rarely skin tension

 Insensitive, without skin tension

**How long can you sunbathe in Germany in early summer (sea level) at midday under a cloudless sky without getting sunburnt?**

 Less than 15 minutes

 Between 15 and 25 minutes

 Between 25 and 40 minutes

 Longer than 40 minutes

**How does your skin react to prolonged sunbathing?**

 Always with a sunburn

 Mostly with a sunburn

 Often with a sunburn

 Rarely or never with a sunburn

**What happens in the event of sunburn?**

 Severe redness, sometimes painful and blistering, followed by peeling of the skin

 Significant redness, after which the skin usually peels

 Redness, after which the skin sometimes peels

 Almost never redness and skin peeling

**Does a tan appear after just one prolonged exposure to the sun?**

 Never

 Mostly not

 Often

 Mostly

**What happens after repeated sunbathing?**

 Hardly any or no tanning at all

 Slight tanning after several sunbathing sessions

 Progressive, more pronounced tanning

 Rapid onset and deep tanning

**What color hair do you (or did you) have?**

 Red to reddish brown

 Light blonde to blonde

 Dark blonde to brown

 Dark brown to black

**What color are your eyes?**
 Light blue, light gray or light green
 Blue, gray or green
 Light brown or dark gray
 Dark brown
